# Clinical predictors of survival in real world practice in stage IV melanoma

**DOI:** 10.1002/cnr2.1691

**Published:** 2022-09-25

**Authors:** Hsien‐Pang Hu, Christine Archer, Desmond Yip, Geoffrey Peters

**Affiliations:** ^1^ ANU Medical School Australian National University Canberra Australia; ^2^ Department of Medical Oncology The Canberra Hospital Canberra Australia; ^3^ College of Nursing & Health Sciences Flinders University Adelaide South Australia Australia

**Keywords:** American Joint Committee on Cancer (AJCC), immunotherapy, melanoma, neutrophil‐to‐lymphocyte ratio, prognostic factors, targeted therapy

## Abstract

**Background and Aim:**

While studies continually identify new clinical prognostic factors in stage IV melanoma, the introduction of targeted and immunotherapies have revolutionised the prognosis of advanced melanoma since 2011. The study aims to investigate the prognostic significance of past and newly identified clinical factors in a contemporary cohort.

**Methods:**

A retrospective analysis of The Canberra Hospital melanoma database identified 161 patients with Stage IV melanoma between 2011 and 2017. Survival was analysed by demographics and clinical factors with chi‐square tests to determine significance. Logistic binary regression was performed to test the independence of the clinical factors on predicting the survival outcome.

**Results:**

Overall, the 3‐month, 6‐month, 9‐month, and 12‐month stage IV melanoma survival rate of our cohort was 79%, 67%, 55%, and 45%, respectively. Age, sex, and BRAF mutation status were found to have no impact on survival, whereas M1d category of the American Joint Committee on Cancer (AJCC) staging (8th edition), neutrophil‐lymphocyte ratio (NLR) >3, elevated serum LDH, more than three metastatic sites, brain metastases, poorer Eastern cooperative oncology group (ECOG) status were associated with poorer survival. Binary logistic regression test identified AJCC staging, NLR (cutoff score 3), LDH, and brain metastases as independent prognostic factors.

**Conclusion:**

Most clinical factors investigated in this study were found to have a statistically significant impact on survival, with AJCC (8th edition) staging M1a‐M1d, NLR (cutoff score 3), LDH, and brain metastases identified as independent prognostic factors in stage IV melanoma from a contemporary cohort treated with targeted therapies and immunotherapies.

## 1 INTRODUCTION (BACKGROUND)

Early studies demonstrated that patients with advanced melanoma have a median survival duration of 6–8 months, with an overall 5‐year survival rate of 2%–6%.^1^, ^2^, ^3^ Since 2011 the landscape of melanoma has changed with new treatments, including targeted therapies such as BRAF inhibitors (dabrafenib and vemurafenib), and MEK inhibitors—(trametinib and cobimetinib), as well as immunotherapies including anti‐CTLA‐4 antibody (ipilimumab), and anti‐PD‐1 antibodies (pembrolizumab and nivolumab). The median survival of advanced melanoma has now increased to approximately 1–2 years, and the overall 5‐year survival also increased to 15%–23%.^4^, ^5^, ^6^, ^7^, ^8^ These novel therapies, either in combination or as monotherapy, provide significant survival improvements compared with traditional cytotoxic chemotherapies.^4^, ^6^, ^9^, ^10^, ^11^, ^12^ However, numerous studies had shown that the prognosis of melanoma, as well as treatment outcomes, are also influenced by a number of clinical factors, including site and number of metastases, serum lactate dehydrogenase (LDH) level, neutrophil‐to‐lymphocyte ratio (NLR), and Eastern Cooperative Oncology Group (ECOG) performance status of the patient.^1^, ^6^, ^13^, ^14^, ^15^, ^16^, ^17^, ^18^, ^19^, ^20^

Melanoma can metastasize both haematogenously and lymphatically with rapid systemic dissemination to distant vital organs that are critical for patient's survival.^17^ Brain metastasis, for example, is very common in Stage IV melanoma, with approximately 50% of patients developing brain metastases during their disease course.^6^, ^13^, ^17^, ^21^ This is associated with an extremely poor prognosis, with a median overall survival of approximately 3 months.^6^, ^13^, ^17^, ^21^ Other sites of common visceral metastases also include lung, liver, and small bowel. The American Joint Commission on Cancer (AJCC) developed the AJCC melanoma staging system classifying Stage IV melanoma into four subcategories ‐ M1a, M1b, M1c, and M1d based on the sites of metastases.^22^ Early studies reported M1c—visceral metastases, typically had the worst prognosis, followed by M1b—lung metastasis without other visceral metastases, and M1a‐ distant skin, subcutaneous, or nodal metastases without other visceral metastases.^16^, ^17^ In 2017, adjustments were applied to the AJCC staging system, and M1d‐ brain metastases with or without other visceral metastases, was added as a new category separated from the previous M1c group and predicted to have a poorer prognosis.^22^

Furthermore, studies have also demonstrated that patients who have less than 3 metastatic sites of disease and normal baseline serum LDH predict a better survival outcome with targeted treatments such BRAF and MEK inhibitors, and thus, a better prognosis.^12^, ^18^ Elevated serum LDH is a well‐known prognostic factor associated with poor survival outcomes of melanoma as well as many other cancers, including, colorectal, lung, breast, prostate, and germ cell cancer, and has been recently reported as a strong predictive marker for poorer response with immunotherapies.^13^, ^14^, ^15^, ^16^, ^23^ Recently, there is increasing evidence indicating NLR, a biomarker of inflammatory status (calculated by dividing the number of circulating neutrophils with lymphocyte counts), has a prognostic value for the survival of melanoma, especially with those patients treated with immunotherapies.^19^, ^24^, ^25^ Studies have reported that high NLR was predictive of poor overall survival, and NLR >3 showed poorer response to immunotherapies in patients with melanoma.^24^, ^25^, ^26^, ^27^ However, the findings were also suggested to be inconclusive, as the cut‐off point varied between studies and treatments.^19^ ECOG, a scale measuring performance status has also been a useful prognostic indicator.^17^, ^20^ Studies have reported that better ECOG, along with female gender and young age, typically showed better melanoma prognosis.^17^, ^20^ In addition, though still disputable, several studies suggested BRAF status—a mutation occurring in approximately 50% of melanoma cases, could be a prognostic factor, as BRAF positive melanomas were typically more aggressive than those BRAF negative.^28^, ^29^, ^30^

In summary the literature findings show advanced AJCC stage, the presence of brain metastasis, the number of metastatic sites greater than 3, high NLR ratio, elevated serum LDH, male with older age and poorer ECOG status, BRAF positive are likely to convey worse survival outcomes. This paper aims to investigate the correlation between these clinical prognostic features and the survival outcomes in a real‐world cohort of patients.

## 2 MATERIALS AND METHODS

### 2.1 Study design

The study is a retrospective analysis of data collected from medical records from The Canberra Hospital (TCH) and Calvary Public Hospital Bruce (CPHB) in Canberra, Australian Capital Territory (ACT), Australia. TCH and CPHB are the major tertiary and secondary public hospitals in Canberra, with 672 and 250 beds, respectively, catering to a population of around 550 000, which includes those from regional New South Wales (NSW). The melanoma database maintained by TCH Department of Medical Oncology, records all the patients who received consultations or treatments for melanoma. Eligibility of patients for the study was first determined based on their stage of melanoma recorded in the melanoma database. Further data collection was done via accessing Australian electronic medical record systems including CHARM, Clinical Portal, CIS information systems, and Capital Pathology by using unique patient identifiers. The study was approved by the Calvary Public Hospital Bruce Human Research Ethics Committee on 8/Nov/2018 and ANU Human Research Ethics Committee on 25/Sept/2019. All the data were first recorded in Microsoft Excel and subsequently analysed by the statistical software‐ IBM SPSS Statistics.

### 2.2 Inclusion and exclusion criteria

Patients with Stage IV metastatic melanoma, de novo or developed from pre‐existing lesions, were included in the study. Those patients with cutaneous, mucosal, and melanoma of unknown primary were included, but ocular melanomas were excluded. We restricted our patient population to those who were diagnosed with Stage IV melanoma between 2011 and 2017. However, patients who diagnosed with Stage IV melanoma before 2011 were also included if they remained active after 2012. Patients were required to have at least 1 year of follow up unless they had died within a year after the initial date of stage IV melanoma diagnosis. Patients without a clear date of diagnosis, or regular follow up scans, and blood test measurements were excluded. Patients who were deceased but without the date of death were also excluded. For the patients who remained active, the censoring date was 8/Nov/2018 which was the date we received ethics approval and commenced data collection.

### 2.3 Data collection and statistical analysis

The data collected included: (i) gender and age, (ii) date of stage IV melanoma diagnosis, (iii) AJCC Staging 8th edition, (iv) number of metastatic sites, (v) serum LDH level and NLR, (vi) ECOG performance status at the time of diagnosis, (vii) date of death for the deceased patients.

The initial date of diagnosis was determined as the date stage IV disease was radiologically confirmed along with histological result of metastatic melanoma. Both the AJCC stage and number of sites metastasis were determined by reviewing the initial radiological assessment (PET‐CT or CT) that confirmed the diagnosis. Patients were stratified based on AJCC 8th edition staging system for melanoma. All radiological imaging was interrogated and the incidence of brain metastases during their disease course was noted.

Pathology testing including serum LDH, neutrophils, and lymphocytes level were collected from the blood test closest to confirmed Stage IV melanoma diagnosis prior to treatment. The NLR ratio was calculated by dividing neutrophil count with lymphocyte count.

The ECOG performance status was collected from the clinical notes recorded in CHARM or deduced by the medical oncologist and skin cancer specialist nurse at TCH based on the descriptions of the patient's status.

BRAF mutation was classified into three categories: (i) BRAF positive—which included V600E, V600K, and V600R, (ii) BRAF negative—absence of BRAF mutation or non‐druggable BRAF mutations, (iii) not performed.

The treatment groups were classified into five categories: (i) patients received targeted therapy but no immunotherapy, (ii) patient received immunotherapy but no targeted therapy, (iii) patients received both targeted and immunotherapy (irrespective of sequence), (iv) patients received no targeted or immunotherapy but had received other therapies, including chemotherapy, surgical metastasectomy, or radiotherapy, (v) patients received no treatments or palliative‐care treatment alone. Immunotherapies included in this cohort were ipilimumab, pembrolizumab, or nivolumab, either in combination or monotherapy. Targeted treatments included were dabrafenib, vemurafenib, trametinib or cobimetinib, either in combination or monotherapy.

The survival duration was calculated in months (from the initial date of diagnosis with Stage IV melanoma to date of death), and for the active patients, the censoring date was 8/Nov/2018. We presented stage IV melanoma survival rate in 3‐month, 6‐month, 9‐month, and 12‐month by demographics and clinical factors listed above. Chi‐square tests were performed to test differences of frequencies and statistical significance. Factors that identified to have a significant impact on survival by chi‐square were subsequently selected and performed logistic binary test, to assess whether each individual factor served as an independent prognostic factor. Kaplan–Meier survival curves were also generated to present the survival pattern of the independent prognostic factor identified by the binary logistic regression test.

## 3 RESULTS

A total of 161 patients were identified for this analysis from TCH melanoma database. The dates of diagnosis with stage IV melanoma were between 28/May/2010 and 3/Nov/2017. The majority of the patients were deceased at the time of collection, with only 38 (24%) reported active at the censoring date. The median overall survival duration of our cohort was 9 months (95%CI = 6.24–11.8 months).

**FIGURE 1:**
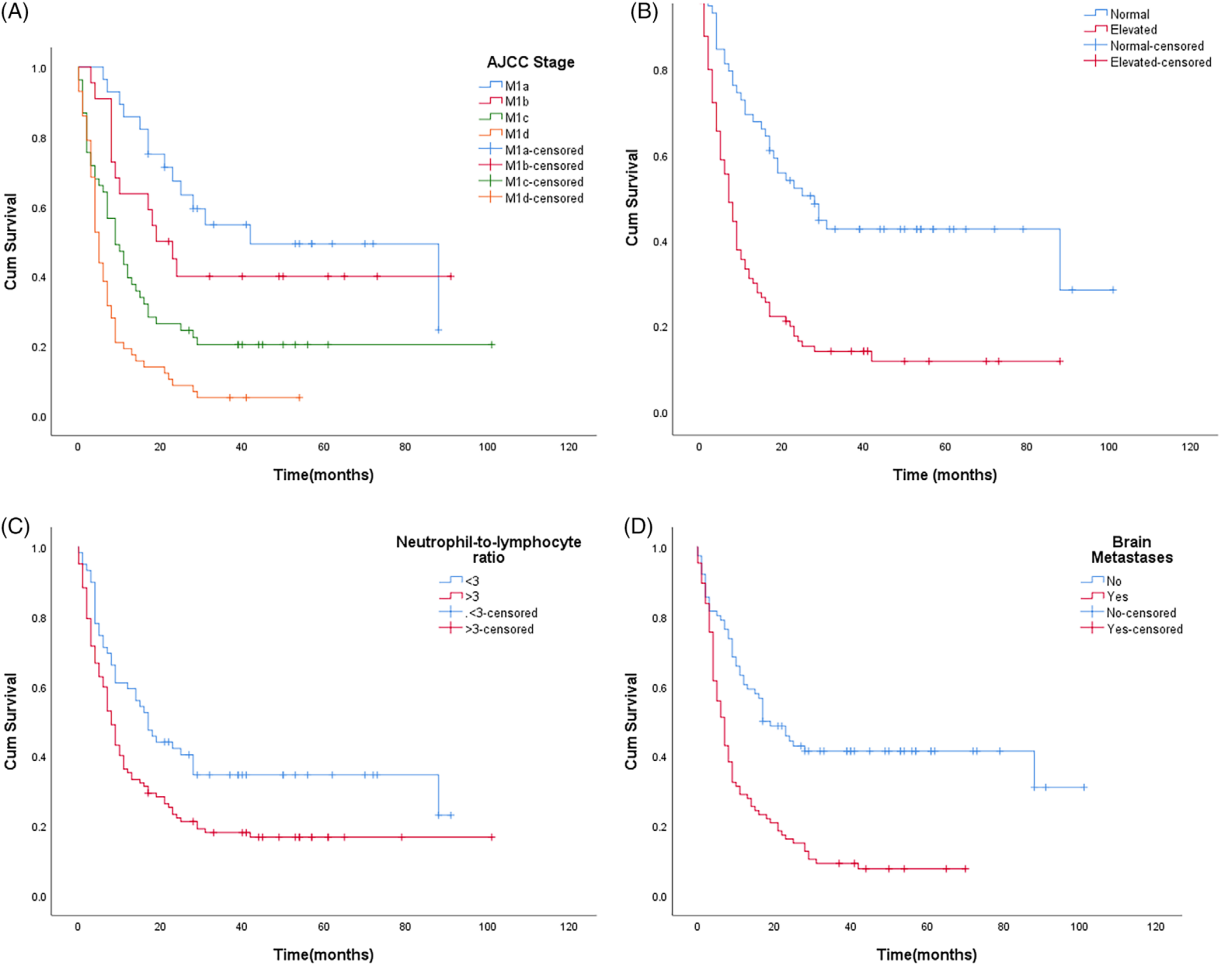
Kaplan–Meier overall survival curves for (A) AJCC stage (*n* = 161), (B) serum LDH (*n* = 148), (C) Neutrophil‐to‐lymphocyte ratio (*n* = 161), and (D) Brain metastases (*n* = 161)

Patient characteristics are summarised in Table 1, and stage IV melanoma‐specific survival rates by demographic and clinical factors are shown in Table 2. Tables 3 and 4 describe the result of the binary logistic test, while Figure 1 illustrates the Kaplan–Meier survival curves of different factors.

**TABLE 1:** Characteristics of the patients, according to study cohort

Characteristic	No. of patients	Percentage (%)
Overall	161	
Age at diagnosis, years		
Median age (range), years	68 (22–84)	
<39	8	5
40–64	58	36
≥65	95	59
Sex		
Male	101	63
Female	60	37
AJCC 8th edition stage^a^		
M1a	27	17
M1b	23	14
M1c	54	34
M1d	57	35
Number of metastatic sites^a^		
Median	2	
≥3	75	46
<3	86	53
Brain metastases during the disease course		
Yes	85	53
No	76	47
Serum LDH level^a^		
Elevated	90	56
Normal	59	37
Missing	13	8
NLR^a^		
Median	3.78	
>3	102	63
<3	59	37
BRAF status^b^		
Positive	57	35
Negative	96	60
Not done	8	5
ECOG^a^		
0	34	21
1	59	37
2	28	17
3	6	4
Missing	34	21
Treatment^c^		
Immunotherapies (IO)	73	45
Targeted therapies	30	19
Both (IO and targeted)	23	14
Other treatments	24	15
No treatment	11	7

*Note*: No treatments group included patients who declined treatment or received with only palliative treatment.

Abbreviations: AJCC, denotes American Joint Committee on Cancer; ECOG, Eastern Cooperative Oncology Group; LDH, lactate dehydrogenase; NLR, neutrophil‐to‐lymphocyte ratio.

^a^ At the initial diagnosis with Stage IV melanoma.

^b^ The BRAF positive included V600E, V600K, and V600R. BRAF negative included those with no BRAF mutation +1 case detected with K601E mutation.

^c^ Immunotherapies treatments included ipilimumab, pembrolizumab or nivolumab, either in combination or monotherapy. Targeted treatments included with dabrafenib, vemurafenib, trametinib or cobimetinib either in combination or monotherapy. Both (IO and targeted)—referred to the case which experienced both immunotherapies and targeted therapies. Other treatments referred to patients who received no targeted or immunotherapy, but received other therapies, including chemotherapy, surgical metastasectomy or radiotherapy.

**TABLE 2:** Stage IV melanoma‐specific survival rate (%) by demographics and clinical factors

Characteristic	3 months	Chi‐square *p*‐value	6 months	Chi‐square *p*‐value	9 months	Chi‐square *p*‐value	12 months	Chi‐square *p*‐value
Overall	79%		67%		55%		45%	
Age at diagnosis, years								
<39	100%	.092	75%	.243	50%	.195	50%	.321
40–64	83%		64%		53%		43%	
≥65	84%		68%		57%		46%	
Sex								
Male	88%	.448	70%	.729	59%	.906	49%	.926
Female	78%		62%		48%		40%	
AJCC 8th edition stage^a^								
M1a	100%	.003*	100%	<.001*	96%	<.001*	89%	<.001*
M1b	100%		87%		70%		61%	
M1c	76%		67%		57%		44%	
M1d	79%		44%		28%		19%	
Number of metastatic sites^a^								
≥3	73%	<.001*	49%	<.001*	39%	<.001*	29%	<.001*
<3	94%		83%		70%		59%	
Brain metastases								
Yes	84%	.751	55%	.001*	39%	<.001*	29%	<.001*
No	86%		80%		74%		63%	
Serum LDH level^a^								
Elevated	80%	.01*	59%	.001*	44%	<0.00*1	33%	<.001*
Normal	95%		85%		76%		69%	
Neutrophil‐to‐Lymphocyte ratio (NLR)^a^								
>3	79%	.02*	63%	.1	49%	.036*	36%	.002*
<3	93%		75%		66%		61%	
BRAF status								
Positive	91%	.074	75%	.084	56%	.761	39%	.235
Negative	80%		61%		54%		49%	
ECOG^a^								
0	97%	<.001*	76%	<.001*	68%	<.001*	53%	<.001*
1	92%		80%		69%		59%	
2	79%		43%		36%		25%	
3	33%		17%		0%		0%	
Treatment^a^								
Immunotherapies (IO)	86%	.001*	73%	<.001*	66%	<.001*	60%	.002*
Targeted therapies	93%		70%		47%		37%	
Both (IO and targeted)	100%		91%		78%		48%	
Other treatments	67%		46%		29%		25%	
No treatments	55%		18%		18%		9%	

^a^ Factors likely have an impact on survival outcome based on Chi‐square findings.

* *p*‐value by Chi‐Square <.05.

**TABLE 3:** Test of independent prognostic factor (AJCC 8th edition stage)

Clinical factors	*p*‐Value
Number of metastatic sites	.466
LDH^a^	.005*
AJCC Version 8 stage^a^	.01*
NLR^a^	.001*
ECOG	.076
Treatment	.123

^a^ The independent prognostic factor identified based on the result of binary logistic regression.

* *p*‐value by binary logistic regression <.05.

**TABLE 4:** Test of independent prognostic factor (Brain metastases)

Clinical factors	*p*‐value
Number of metastatic sites	.073
LDH^a^	.004*
Brain metastases^a^	.001*
NLR^a^	.004*
ECOG	.109
Treatment	.082

^a^ The independent prognostic factor identified based on the result of binary logistic regression.

* *p*‐Value by binary logistic regression <.05.

### 3.1 Patient demographics and clinical characteristics

The median age was 68 years (range: 22–84), with 95% of patients over age of 40. Hundred one (63%) subjects were male. About 60% were BRAF negative with 1 case identified with the rare K601E mutation, and 5% of patients did not have the BRAF mutation test. M1d was the predominant AJCC stage, accounting for 35% of our cohort, followed by M1c (34%), M1a (17%) and M1b (14%). In total, 53% of patients developed brain metastases during their disease course. The median number of metastatic sites was 2, with 53% of subjects reporting less than three metastatic sites. Thirteen (8%) patients did not have serum LDH available. Of the remaining subjects, 90 (56%) patients were identified with elevated LDH and 59 (37%) with normal LDH. The median of NLR was 3.78, with (63%) detected with NLR higher than 3. 34 (21%) patients did not have their ECOG recorded and could not be deduced based on their clinical notes. The remaining subjects were predominately ECOG 1 (37%), followed by ECOG 0 (21%), ECOG 2 (17%), and ECOG 3 (4%). Lastly, immunotherapies were the predominant treatment used in this cohort, with a total of 59% of patients receiving checkpoint inhibitors (45% of those with immunotherapies only and 14% with both targeted and immunotherapies) (Table 1).

### 3.2 Survival rate and prognostic factor analysis

Overall, the 3‐month, 6‐month, 9‐month, and 12‐month stage IV melanoma survival rate of our cohort was 79%, 67%, 55%, and 45%, respectively. The survival rate by demographics and clinical factors are summarised in Table 2, and chi‐square tests were performed to test the significance of the difference. Our finding suggests that age, sex, and BRAF were unlikely to have an impact on survival outcome, with no statistical difference reported across 3, 6, 9 and 12‐month survival (Table 2). AJCC stage M1a, M1b, M1c, and M1d, number of metastatic sites, brain metastases, serum LDH level, NLR, ECOG, and treatment, all suggested having a statistically significant impact on survival outcome based on the findings of chi‐square analysis (Table 2).

M1a patients demonstrated the highest 3‐month (100%), 6‐month (100%), 9‐month (96%) and 12‐month (89%) survival, followed by M1b (100%, 87%, 70%, and 61%). M1d had a slightly better 3‐month survival (79%) than the M1c (76%), but in general M1c had better survival compare to M1d, with higher survival rate in 6‐month (67% vs. 44%), 9‐month (57% vs. 28%), and 12‐month (44% vs. 19%) (Table 2). The chi‐square *p*‐values of AJCC stage in 3, 6, 9 and 12‐month survival were all significant (.003, <.001, <.001, and <.001 respectively).

Number of metastatic sites less than three had better survival in 3‐month (94%), 6‐month (83%), 9‐month (70%), and 12‐month (59%) compare to cases with more than three metastatic sites (73%, 49%,39%, and 29% respectively), and the differences were all statistically significant with *p*‐values all reported with <.001 (Table 2). Brain metastases had no statistically significant impact on survival within 3 months (*p*‐value = .751), however showed poorer survival outcomes beyond 3‐months, with survival rates significantly lower in 6, 9, and 12‐months compare to cases with no brain metastases (Table 2).

Subjects with elevated serum LDH level had poorer 3‐month (80%), 6‐month (59%), 9‐month (44%), and 12‐month (33%) compare to subjects with normal serum LDH (86%, 80%, 74%, and 63%, respectively), and the differences were all statistically significant (*p*‐value: .01, .001, <.001, <.001, respectively) (Table 2).

NLR of more than three had poorer 3‐month (79%), 6‐month (63%), 9‐month (49%), and 12‐month (36%) survival rates compared to NLR less than 3 (93%, 75%, 66%, and 61%, respectively), although the difference in 6‐month survival was not significant (*p*‐value =0.1). The differences in 3,9, and 12‐month survival were all statistically significant (*p*‐value: .02, .036, and .002, respectively).

Patients with ECOG 3 had the worst 3‐month (33%), 6‐month (17%), 9‐ month (0%) and 12‐month (0%) survival, followed by ECOG 2 (79%, 43%, 36%, and 25% respectively). The chi‐square *p*‐values of ECOG in 3, 6, 9 and 12‐month survival rate were all significant with *p*‐value <.001 (Table 2). Mix findings were reported between ECOG 0 and ECOG 1, with ECOG 0 had better 3‐month survival than ECOG 1(97% vs. 92%), but poorer 6‐month (76% vs. 80%), 9‐month (68% vs. 69%), and 12‐month survival (53% vs.59%) (Table 2).

Similar to the ECOG, the chi‐square test suggested treatment as a whole had significant impact on in 3, 6, 9, and 12‐month survival (*p*‐value: .001, <.001, <.001, .002, respectively), but less united pattern on showing which subgroup had the best survival outcome (Table 2). However, overall, it appeared the group who received both immunotherapies and targeted therapy during their disease course had the best survival rate (3‐month: 100%, 6‐month: 91%, 9‐month: 78%, and 12‐month: 48%), followed in order by the group receiving only immunotherapies (86%, 73%, 66%, and 66%, respectively), targeted therapies only (98%, 70%, 47%, and 37%, respectively), traditional treatments only (67%, 46%, 29%, and 25%, respectively), and no treatments (55%, 18%, 18%, and 9%, respectively) (Table 2).

Based on the findings of Table 2, we selected the AJCC stage, brain metastases, number of metastatic sites, serum LDH level, NLR, ECOG, and treatment to perform binary logistic regression for independent prognostic factor investigation. However, we tested the AJCC stage and brain metastases separately as these two factors have some overlaps in classification (as M1d also indicated brain metastases). Both binary logistic regression test showed serum LDH, NLR (with cutoff score 3) were independent prognostic factors (Tables 3 and 4). Both the AJCC stage and brain metastases were found to be capable of predicting the survival outcome of stage IV melanoma independently (Table 3 and Table 4). The Kaplan–Meier overall survival curves further illustrated the pattern of each factor, and the findings all corresponded to the survival trends described in Table 2, with advanced AJCC stage, Elevated LDH, High NLR and the presence of brain metastasis reported with poorer survival outcome.

## 4 DISCUSSION

Our study included 161 cases of stage IV melanoma diagnosed between 2010 and 2017 at TCH. The median overall survival duration of our cohort was 9 months (95%CI = 6.24–11.8 months), with 123 (76%) cases deceased at the time of data collection. Our samples were predominately males, while the median age was 68, ranging between 22 and 84, with 95% of the cohort over the age of 40. Overall, these demographic findings did correspond to the worldwide and Australia epidemiological trends reported, which indicated melanoma was more common in male and age group over 40.^31^, ^32^

We distributed the Stage IV melanoma incidence by demographics and clinical factors and by 3, 6, 9, and 12‐month melanoma‐specific survival data (Table 2). Overall, the 3, 6, 9, and 12‐month survival rates were 79%, 67%, 55%, and 45%, respectively. Our cohort's median overall survival duration and 12‐month survival rate were similar to the findings of studies prior to or around 2011, in which targeted and immunotherapies were emerging or not yet widely available, with estimated median overall survival length of 6–9 months and 12‐month survival rate of 40%–45%.^1^, ^2^, ^3^, ^14^, ^17^ However, since 78% of our cases received novel treatments introduced after 2011, our overall survival results were lower than we expected. This was likely associated with high prevalence of brain metastases at the time of diagnosis, accounting for 53% of our cohort. However, this might also be due to the fact that the management of stage IV melanoma has only just been revolutionised within the last 5 years, that most of the novel treatments were not available to patients on Australian Pharmaceutical Benefits Scheme (PBS) until 2015.^33^ Many of the novel treatments were only accessible on clinical trial (unavailable in Canberra) at the time our patients were diagnosed with Stage IV melanoma, and thus, many of our patients only received these treatments at a later stage of their disease course.

In general, our findings on prognostic factors were mostly in line with past studies, showing that advanced AJCC stage, number of metastatic sites >3, brain metastases, elevated serum LDH level, NLR >3, and poorer ECOG status, were associated with poor survival outcomes.^1^, ^6^, ^13^, ^14^, ^15^, ^16^, ^17^, ^18^, ^19^, ^20^, ^27^ However, we did not find any statistically significant difference in melanoma‐specific survival in different age groups, sex, and BRAF status, which early studies typically reported male, age over 65, and BRAF positive as factors associated with poorer prognosis of melanoma.^1^, ^6^, ^13^, ^14^, ^15^, ^16^, ^17^, ^18^, ^19^, ^20^ Since our cohort was predominately male (63%), age over 65 (59%), and BRAF negative (60%), the relatively small and unequally distributed sample size may be a primary reason of the statistical non‐significance. However, in contrast to earlier stages (I‐III) of melanoma, where age and sex are generally accepted as independent prognostic factors in past studies, the prognostic significance of age and sex is more conflicting in studies involving stage IV melanoma.^34^, ^35^, ^36^ While studies reported mixed findings on the existence of female survival advantage in stage IV melanoma, the results on whether the female survival advantage persists across age groups were also inconclusive.^34^, ^35^, ^36^ The inconsistency of prior studies may be attributed to the different selection of prognostic variables and different staging systems and treatments influenced by the study year.^35^ Multiple studies found that the significance of female survival advantage decreased or disappeared when considering the increased number of metastatic sites.^34^, ^36^, ^37^ It is also important to note that most of these studies were conducted on cohorts prior to 2011, when immunotherapies and targeted therapies were not available. A recent study conducted in a contemporary patient cohort with novel therapies, staged with the latest 8th edition AJCC staging system, found that while female survival benefits existed across all stages (I–IV) of melanoma, it was widely influenced by patient's age and most significant in women age <45.^35^

Similarly, while BRAF mutation status in metastatic melanoma was commonly found to associate with factors that indicate worse survival, such as higher AJCC stages and metastasis at a younger age, the prognostic impact of BRAF status seemed controversial in previous studies, especially considering the influence of other factors such as ECOG, metastatic sites and LDH.^30^, ^38^ Conflicting results were also likely due to potential selection bias in some retrospective analyses. In order to investigate the treatment‐unrelated impact of BRAF mutation, these studies typically only selected BRAF patients excluded from clinical trials for targeted inhibitors due to having factors such as elevated LDH, brain metastasis or poor ECOG, leading a patient cohort biased towards worse prognosis.^38^ Nevertheless, the application of the novel treatments in our cohort could likely reduce the prognostic influence of BRAF status, as studies reported both targeted and immunotherapies significantly improved the survival of BRAF‐positive patients.^10^, ^11^, ^39^, ^40^

Further analysis via binary logistic regression identified serum LDH, NLR (cutoff score 3), brain metastases, AJCC stage (8th edition) as independent prognostic factors. The prognostic value of serum LDH and brain metastases were widely identified by past studies and studies in contemporary cohort with novel treatments.^6^, ^9^, ^13^, ^14^, ^15^, ^21^, ^35^ AJCC stage 8th edition that added the new M1d category separated from the AJCC stage 7th M1c subgroup, was only recently published and with relatively limited studies.^22^ Our results showed a similar pattern as reported in studies on the earlier version of AJCC, with M1a stage conveying the best prognosis, followed by M1b, and M1c; whilst our study also supported AJCC stage 8th, with M1d replaced M1c, becoming the new AJCC subgroup with the worst survival outcome.^16^, ^17^, ^23^

Our findings supported previous studies which reported NLR >3 as an independent prognostic factor to predict poor prognosis in melanoma.^19^, ^24^, ^25^, ^26^, ^27^ Our results, however, differed from past studies which only evaluated patients who received immunotherapies, whilst our study included patients who received other treatment types, suggesting the prognostic value of NLR is not limited to immunotherapy. Since the majority (59%) of our cases received immunotherapies, further investigation is still required to confirm the prognostic value of NLR in targeted therapies as well as other therapies. In fact, past studies also found that NLR cutoff score 3 could be an independent prognostic factor in metastatic melanoma treated with chemotherapy, as well as non‐metastatic melanoma treated surgically.^27^, ^41^

Apart from being a retrospective study with a small sample size, our analysis is also subjective to other limitations. In total, 47% of our patients did not have an ECOG record in CHARM. While we were able to deduce 26% of these cases' ECOG based on the descriptions of the patient's status from clinical notes, this poses risks to potential information and selection bias, and 21% of patients still miss an ECOG record due to a lack of information. Another important consideration is that our analysis simplified our treatment variations by categorising them into major groups. We did not distinguish different types of immunotherapies or targeted therapies, either in monotherapies or combination therapies as well as with or without surgical metastasectomy or radiotherapy, nor evaluated the sequence of different treatments given, which past studies had reported variations in survival outcomes.^42^, ^43^, ^44^, ^45^, ^46^, ^47^, ^48^ Furthermore, while we excluded ocular melanoma, which is highly resistant to systemic therapies and typically treated differently, we did not further analyse the differences in prognosis among other melanoma subtypes due to our limited sample size and data regarding the primary tumours. We also did not differentiate de novo stage IV melanoma from stage IV melanoma with an initial diagnosis from an earlier stage, which could potentially cause variations in prognosis as well as treatments exposed. Ideally, future investigations should include a larger sample size, with further stratification into independent subgroups for comprehensive observation between clinical factors and treatments on survival outcomes of advanced melanoma.

## 5 CONCLUSION

Our study support previous findings that higher AJCC stages, presence of brain metastases, NLR >3, number of metastatic sites >3, elevated LDH, and poorer ECOG, are all associated with poorer prognosis of stage IV melanoma. Further analysis demonstrated the AJCC (8th edition) stage, NLR (cutoff score 3), serum LDH, and brain metastases could be independent prognostic factors for stage IV melanoma survival in a contemporary cohort treated with novel therapies. The addition of M1d classification in AJCC stage (8th edition) is also validated in our study, as M1d showed significantly worse survival outcomes than M1c. While our findings suggest the prognostic value of NLR may not limited to immunotherapies, further research is needed to determine whether this could be used as an independent prognostic factor for patients receiving other treatments.

## AUTHOR CONTRIBUTIONS

**Hsien‐Pang Hu:** Formal analysis (equal); investigation (lead); methodology (equal); writing – original draft (lead). **Christine Anne Archer:** Data curation (supporting); project administration (supporting); writing – review and editing (supporting). **Desmond Yip:** Supervision (equal); writing – review and editing (supporting). **Geoffrey Peters:** Conceptualization (lead); data curation (equal); methodology (supporting); project administration (equal); supervision (equal); writing – review and editing (equal).

## CONFLICT OF INTEREST

Geoffrey Peters has received honoraria from BMS and MSD. Christine Archer has received honoraria from BMS and MSD.

## ETHICS STATEMENT

Assessed by The ACT Health Human Research Ethics Committee's Low Risk Sub‐Committee—deemed no approval required due to negligible risk to participants (date: 24/Oct/2018, QAI.00194). Ethics approval granted from Calvary Public Hospital Bruce Human Research Ethics Committee on 08/Nov/2018 with ethics approval number 27–2018. Ethics approval granted from ANU Human Research Ethics Committee on 25/Sept/2019 with ethics approval number 2019/759.

## Data Availability

The data that support the findings of this study are available on request from the corresponding author. The data are not publicly available due to privacy or ethical restrictions.
